# P-1660. Longitudinal Single-Center Study of Anti-RBD IgG Responses Among Healthcare Workers During Different SARS-CoV-2 Variant Waves

**DOI:** 10.1093/ofid/ofaf695.1835

**Published:** 2026-01-11

**Authors:** Vilija Gurksniene, Tadas Alcauskas, Fausta Majauskaite, Jurgita Urboniene, Ligita Jancoriene

**Affiliations:** Vilnius University, Vilnius, Vilniaus Apskritis, Lithuania; Vilnius University Faculty of Medicine, Vilnius, Vilniaus Apskritis, Lithuania; Clinic of Infectious Diseases and Dermatovenerology, Institute of Clinical Medicine, Faculty of Medicine, Vilnius University, Vilnius, Vilniaus Apskritis, Lithuania; Vilnius University Hospital Santaros Clinics, Vilnius, Vilniaus Apskritis, Lithuania; Clinic of Infectious Diseases and Dermatovenerology, Institute of Clinical Medicine, Faculty of Medicine, Vilnius University, Vilnius, Lithuania, Vilnius, Vilniaus Apskritis, Lithuania

## Abstract

**Background:**

It is evident that different SARS-CoV-2 variants can elicit varying humoral immune response. This response can be influenced by mutations in the virus's spike protein and other structural proteins. The objective of this study was to evaluate the dynamics of receptor-binding domain-specific antibodies (anti-RBD IgG) following COVID-19 vaccination during periods characterized by the prevalence of different SARS-CoV-2 variants.

**Figure:**
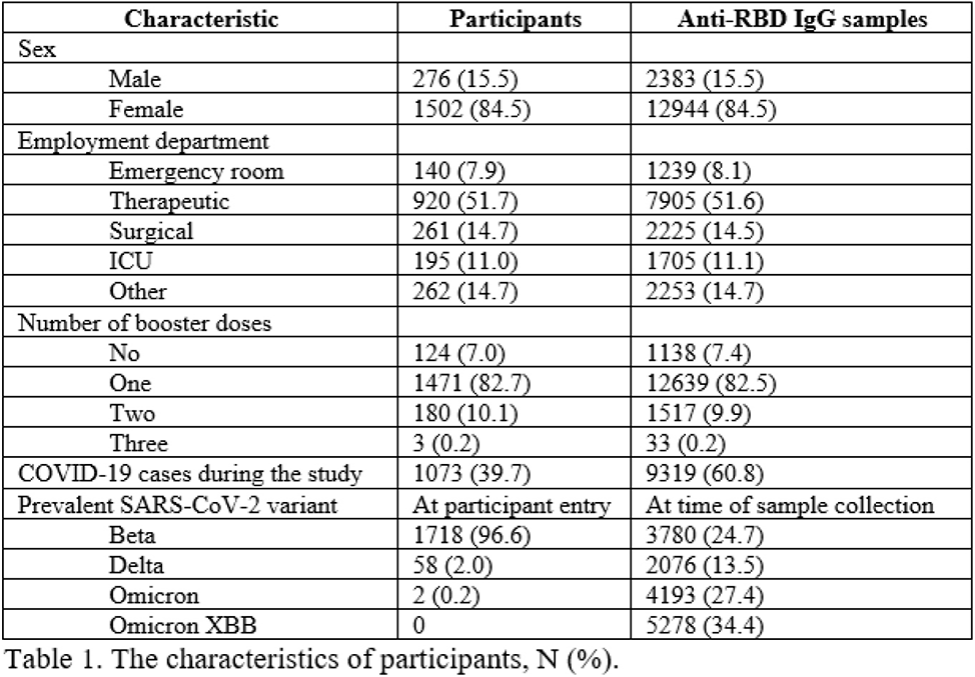

**Figure:**
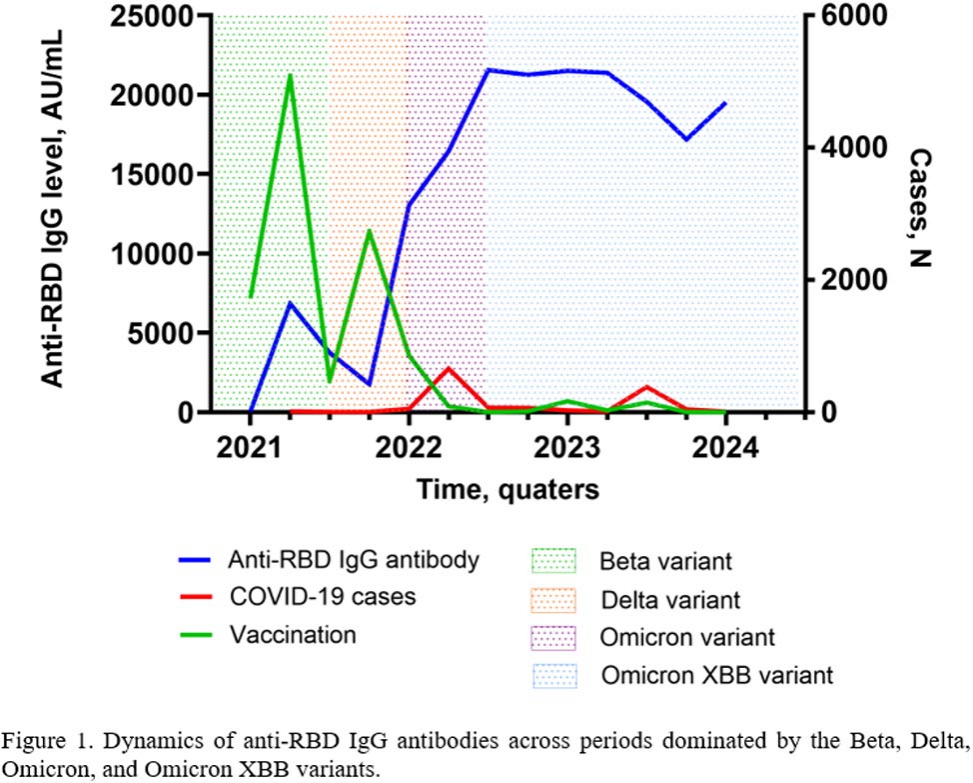

**Methods:**

A prospective observational study was conducted at Vilnius University Hospital Santaros Clinics (VUH SC), Lithuania. Participants were VUH SC employees who had completed a primary vaccination series and provided informed consent. The primary vaccination series was defined as either two doses of a Pfizer-BioNTech or Moderna mRNA vaccine, a single dose of the Janssen vaccine, or a confirmed SARS-CoV-2 infection following one vaccine dose. Blood samples were collected every three months to measure anti-RBD IgG levels. COVID-19 cases were confirmed through PCR testing, rapid antigen tests, or a positive anti-N IgG result (≥1.4 S/C).

**Figure:**
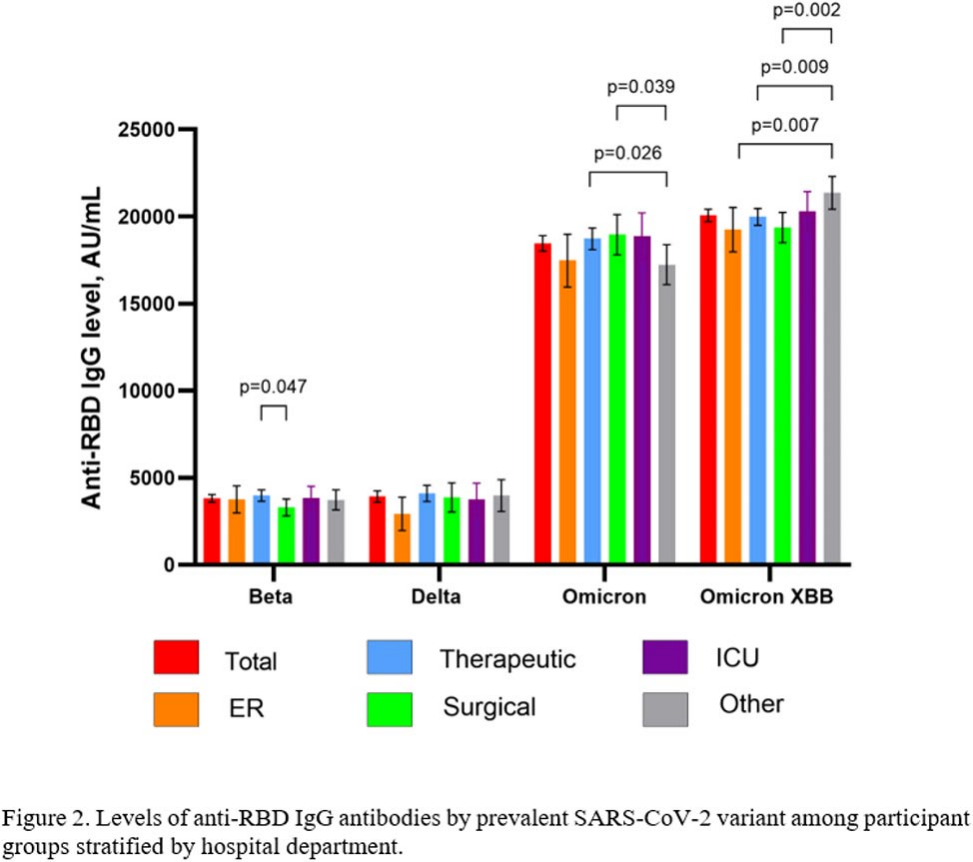

**Results:**

This study included 1,778 participants. Their characteristics are presented in Table 1. The highest number of COVID-19 cases and anti-RBD IgG levels were observed during the spread of the Omicron XBB variant (20069.36 ± 13041.44 AU/mL), significantly higher than those during the Omicron (18458.38 ± 14556.41 AU/mL), Beta (3812.01 ± 6983.83 AU/mL), and Delta (3921.97 ± 7562.29 AU/mL) variant periods (p < 0.001). Following the primary vaccination, anti-RBD IgG levels declined to a nadir of 1,761.2 AU/mL in third quarter (Q3) of 2021. Thereafter, a rise in antibody titers was observed, driven by booster vaccinations and infections with the Omicron and Omicron XBB variants (Figure 1). When comparing antibody titers among employees from different hospital departments, no substantial overall differences were observed. During the Omicron period, the lowest antibody levels were observed among staff from other hospital departments, compared to those in therapeutic and surgical departments (Figure 2).

**Conclusion:**

In the cohort of 1778 healthcare workers, anti-RBD IgG levels varied across SARS-CoV-2 variant periods, peaking during the Omicron XBB wave. Overall differences between hospital departments were minimal.

**Disclosures:**

All Authors: No reported disclosures

